# High-dose thiotepa, in conjunction with melphalan, followed by autologous hematopoietic stem cell transplantation in patients with pediatric solid tumors, including brain tumors

**DOI:** 10.1038/s41409-022-01820-5

**Published:** 2022-11-03

**Authors:** Junichi Hara, Kimikazu Matsumoto, Naoko Maeda, Mariko Takahara-Matsubara, Saori Sugimoto, Hiroaki Goto

**Affiliations:** 1 Department of Pediatric Hematology/Oncology, Children’s Medical Center Osaka City General Hospital, Osaka, Japan; 2 Children’s Cancer Center, National Center for Child Health and Development, Tokyo, Japan; 3 Department of Paediatrics, National Hospital Organization, Nagoya Medical Center, Nagoya, Japan; 4 Sumitomo Pharma Co., Ltd, Osaka, Japan; 5 Division of Hematology/Oncology, Kanagawa Children’s Medical Center, Yokohama, Japan

**Keywords:** Drug development, Chemotherapy

## Abstract

Among pediatric malignancies, solid tumors, particularly within the central nervous system (CNS), are common. Thiotepa, a myeloablative, high-dose chemotherapeutic (HDT) treatment administered prior to autologous hematopoietic stem cell transplantation (HSCT), can cross the blood-brain barrier and rapidly penetrate the CNS. We evaluated thiotepa HDT in conjunction with melphalan in Japanese patients with pediatric CNS/non-CNS solid tumors in a multicenter, open-label, non-comparative study. Thiotepa (200 mg/m^2^/day) was administered intravenously (IV) over 24 h on days −12, −11, −5, and −4 before scheduled HSCT. Melphalan (70 mg/m^2^/day) was administered IV over 1 h on days −11, −5, and −4. The safety analysis population comprised 41 patients, of whom 16 (39.0%) had solid tumors and 25 (61.0%) had brain tumors. The most frequently reported adverse events were diarrhea (40/41 [97.6%] patients) and febrile neutropenia (34/41 [82.9%]). No unexpected safety events were observed, and no events resulted in death or treatment discontinuation. All patients experienced bone marrow suppression and 39/41 (95.1%) achieved engraftment (neutrophil count ≥500/mm^3^ for 3 consecutive days after HSCT). The survival rate at day 100 post-autologous HSCT was 100%. These data confirm the safety of IV thiotepa plus melphalan HDT prior to autologous HSCT for patients with pediatric CNS/non-CNS solid tumors. *Trial registration*: JapicCTI-173654.

## Introduction

Globally, cancer is a major cause of childhood mortality, and incidence rates appear to be increasing over time [1]. Around 2500 children are newly affected by cancer every year in Japan [2], corresponding to an incidence of 1.23/million for ages 0–14 years and 142/million for ages 15–19 years [3].

After leukemia, solid tumors, particularly those occurring within the central nervous system (CNS) are the most common pediatric cancers [4]. In 2015, 904 children with solid tumors were newly registered to the Japanese Society of Pediatric Hematology/Oncology database [5]. High-dose chemotherapy (HDT) and hematopoietic stem cell transplantation (HSCT) allows intensive treatment of such malignancies [6]. Thus, doses of cytotoxic therapies can be escalated beyond marrow tolerance. It is estimated that in Japan, 3 323 children <16 years underwent autologous HSCT for pediatric solid tumors between 1991–2018 [7].

Thiotepa is a myeloablative HDT treatment administered prior to autologous HSCT to treat solid tumors and hematological malignancies [8]. It is an antitumor alkylating agent belonging to the ethyleneimine group, and inhibits DNA synthesis [9]. Importantly, it has the capacity to cross the blood-brain barrier and penetrate rapidly into the CNS, producing concentrations in the cerebrospinal fluid >90% of those observed in serum [8, 10]. Thiotepa has been in clinical use since the 1950s [11, 12], and was approved for standard-dose chemotherapy in Japan in 1958. Its use as HDT prior to HSCT was approved in Europe in 2010 [13]. However, thiotepa was not available for this purpose in Japan, despite the critical medical need, because manufacturing had been discontinued in 2008 [14, 15].

A recent phase I study established the pharmacokinetics of thiotepa as HDT with autologous HSCT in a Japanese population of nine pediatric and 10 adult patients [8]. Treatment was well tolerated and survival rates were high (77.8% for pediatric solid tumors and 100% for malignant lymphoma) [8]. Thiotepa is now approved in Japan as HDT before autologous HSCT for pediatric malignant solid tumors [16, 17].

An expanded access program, including patients with pediatric solid tumors or brain tumors, and patients with malignant lymphoma, was initiated to further evaluate the safety and efficacy of thiotepa as HDT before autologous HSCT. We report here the data from the population with pediatric solid tumors or brain tumors.

## Patients and methods

### Patients

This expanded access study included two groups of patients scheduled to undergo autologous HSCT. This report focuses on patients with pediatric solid tumors or brain tumors; data from the group of patients with malignant lymphoma are reported elsewhere [18].

The key inclusion criteria were patients aged ≥2 years with solid tumors or brain tumors who had completed hematopoietic cell collection for autologous HSCT; Eastern Cooperative Oncology Group (ECOG) performance status (PS) of 0–2 assessed within 14 days before enrollment; negative pregnancy test, and willingness and ability to use appropriate contraception until 90 days after the end of study treatment (if age appropriate); and normal hepatic, renal, and cardiac function based on tests performed within 14 days before enrollment. In addition, the estimated glomerular filtration rate (eGFR), calculated using the levels of serum creatinine or cystatin C measured within 14 days before enrollment was required to be ≥60 ml/min/1.73 m^2^ (patients aged ≥18 years), or ≥100 ml/min/1.73 m^2^ (patients aged <18 years). Each patient (for those aged ≥20 years) and/or their legal representative (for those aged <20 years) provided written informed consent prior to study enrollment.

Key exclusion criteria were patients who had undergone any treatment (other than hematopoietic cell collection) for the underlying disease within 13 days before the start of study treatment; previous HSCT within the 6 months prior to the study; receipt of live attenuated vaccine within 90 days or any investigational agent within 27 days before the start of study treatment; pregnancy or lactation; present or previous history of complications affecting drug metabolism or excretion; active infection; presence of hepatitis B surface antigen or antibody, hepatitis B core antibody, or human immunodeficiency virus antibody (although patients positive for hepatitis B surface antigen or antibody, or hepatitis B core antibody could be enrolled if vaccinated for type B hepatitis); uncontrolled intercurrent illness; hypersensitivity to the study drugs or their excipients; and any other reason which may endanger the patient or confound the study outcomes based on the judgment of the study investigator. Patients who were considered likely to have difficulty in receiving an adequate volume of replacement fluid and frequent blood transfusions (often required in association with concomitant melphalan) were also excluded.

### Study design and treatments

This multicenter, open-label, non-comparative, expanded access program (JapicCTI-173654) was conducted between September 2017 and June 2020 at eight sites in Japan (of which four enrolled patients with malignant lymphoma and four enrolled patients with pediatric solid tumors). Following the approval of thiotepa in Japan as HDT before autologous HSCT for pediatric malignant solid tumors (on March 26, 2019), the study continued as a post-marketing clinical study. The study protocol and related documentation were approved by the ethics committee or independent review board of each study center. The study was conducted in accordance with the Declaration of Helsinki, good clinical practice guidelines, and all applicable legal and regulatory requirements.

**Fig. 1:**
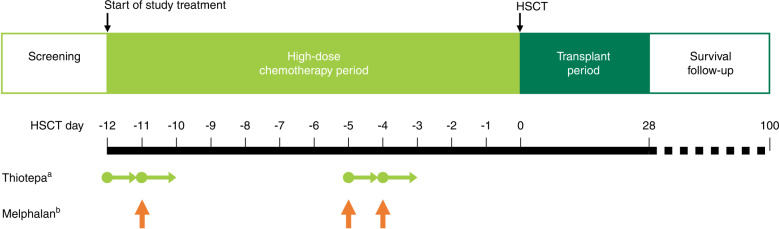
Study design in patients with pediatric solid tumors or brain tumors. The screening period included collection of informed consent and study enrollment. Day 0 was the day of HSCT. ^a^Thiotepa 200 mg/m^2^/day intravenously (IV) over 24 h on days −12, −11, −5, and −4. ^b^Melphalan 70 mg/m^2^/day IV over 1 h on days −11, −5, and −4. *HSCT* hematopoietic stem cell transplantation.

The study design is shown in Fig. 1. The study comprised a HDT period and a transplant period, with the day of HSCT defined as day 0. Thiotepa (200 mg/m^2^/day) was administered intravenously (IV) over 24 h on days −12, −11, −5, and −4 before scheduled HSCT. Commercially available melphalan (70 mg/m^2^/day) was administered IV over 1 h on days −11, −5, and −4 before scheduled HSCT. Melphalan was chosen for the combination treatment regimen in both the phase I study [8] and the current expanded access program based on its regulatory approval status in Japan for pretreatment prior to autologous HSCT in patients with pediatric solid tumors, and its widespread domestic use for this purpose. The doses of thiotepa and melphalan could be reduced or interrupted if deemed necessary by the investigator. Both drugs were to be discontinued if eGFR was <45 ml/min/1.73 m^2^ (patients aged ≥18 years) or <75 ml/min/1.73 m^2^ (patients aged <18 years) on day −7 before scheduled HSCT (i.e., scheduled treatment administration on days −5 and −4 did not occur under these circumstances).

Prohibited concomitant medications and therapies during the study period included any cancer therapy (other than study treatments), any other investigational drugs, and live vaccines.

For these patients with pediatric solid tumors or brain tumors, skin management was recommended to avoid severe dermatologic toxicity such as skin peeling. In addition, as patients were receiving concomitant melphalan, replacement fluid (≥2000 ml/day) and diuretics were supplied to ensure adequate urine volume (≥100 ml/h). Volumes of replacement fluid could be adjusted depending on the age and condition of the patient.

### Endpoints

The primary study objective was to assess the safety of IV thiotepa HDT in combination with melphalan before autologous HSCT in patients with pediatric solid tumors or brain tumors. Safety was assessed by recording treatment-emergent adverse events (TEAEs) and adverse drug reactions occurring between the start of study treatment and day 28 post HSCT. TEAEs were classified using the Medical Dictionary for Regulatory Activities, version 19.1. Ascertainment of causality was undertaken solely by individual treating investigators, without study monitoring or review/modification by the primary investigators. Additional safety evaluations included ECOG PS at each visit, and physical and laboratory test results, including 12-lead electrocardiogram, left ventricular ejection fraction, laboratory measures, vital signs, and weight.

The secondary objective was to assess the clinical outcomes following the use of IV thiotepa in this patient population. Endpoints included the bone marrow suppression rate (defined as the proportion of patients with a neutrophil count <500/mm^3^ at least once during the 28 days after HSCT), the engraftment rate (defined as the proportion of patients with a neutrophil count ≥500/mm^3^ for 3 consecutive days after bone marrow suppression and HSCT), time to engraftment (defined as the number of days between HSCT and the first of three consecutive days with a neutrophil count ≥500/mm^3^ after bone marrow suppression and HSCT), and the survival rate at day 100 post-HSCT.

### Statistical methods

No formal study size calculations or hypothesis testing was performed. The overall sample size for the expanded access program was approximately 100 patients, comprising both those with pediatric solid tumors or brain tumors (reported herein) plus adults with malignant lymphoma (reported elsewhere [18]) based on the expected number of participants.

The safety analysis population included all patients who received at least a single dose of thiotepa. The number and frequency of adverse events and adverse drug reactions were summarized; physical and laboratory test results were reported as summary statistics and electrocardiogram interpretations as shift tables.

For the efficacy analyses of bone marrow suppression rate, engraftment rate and time to engraftment, missing data were not imputed. The rate of survival at day 100 post-HSCT was estimated using Kaplan–Meier methodology. Death after HSCT (regardless of cause of death) was defined as an event, and observations were censored on the latest date of confirmed survival. Statistical analyses were conducted using SAS software version 9.4 (SAS Institute Inc., Cary, NC, USA).

## Results

### Patients

A total of 41 patients with pediatric solid tumors or brain tumors were enrolled into the study. Of these, 38 were enrolled during the expanded access phase and three during the post-marketing phase; data from all 41 patients were summarized together. All patients received thiotepa and were included in the safety analysis population. All 41 patients underwent peripheral blood HSCT.

Baseline demographics and clinical characteristics are shown in Table 1. Overall, 22/41 (53.7%) patients were female, the median age was 4 years, and 31/41 (75.6%) patients were aged between ≥2 and <12 years. Most patients (30/41 [73.2%]) had an ECOG PS of 0. A total of 16/41 (39.0%) patients had a pediatric non-CNS solid tumor, of which the most common was neuroblastoma (5/41 [12.2%]), and 25/41 (61.0%) had a pediatric brain tumor, of which the most common was medulloblastoma (12/41 [29.3%]). Tumors were newly diagnosed in 20/41 (48.8%) patients and relapsed in 21/41 (51.2%) patients; 4/41 (9.8%) patients had received one prior HSCT transplantation and 2/41 (4.9%) had received two or more.

**Table 1:** Baseline demographics and clinical characteristics (safety analysis set).

	Patients with pediatric solid tumors or brain tumors (*N* = 41)
Sex (female), *n* (%)	22 (53.7)
Age (years), median (min, max)	4.0 (2, 31)
≥2 to <12 years, *n* (%)	31 (75.6)
≥12 to <16 years, *n* (%)	4 (9.8)
≥16 years, *n* (%)	6 (14.6)
Height (cm), median (min, max)	101.0 (74.0, 185.0)
Weight (kg), median (min, max)	14.7 (9.3, 88.6)
BSA (m^2^)^a^, median (min, max)	0.6 (0.4, 2.1)
ECOG PS, *n* (%)	
0	30 (73.2)
1	9 (22.0)
2	2 (4.9)
Tumor type, *n* (%)	
Pediatric solid tumor	16 (39.0)
Neuroblastoma	5 (12.2)
Rhabdomyosarcoma	3 (7.3)
Retinoblastoma	3 (7.3)
Malignant rhabdoid tumor	2 (4.9)
Ewing’s sarcoma	1 (2.4)
Nephroblastoma	1 (2.4)
York sac tumor	1 (2.4)
Brain tumor	25 (61.0)
Medulloblastoma	12 (29.3)
Atypical teratoid/rhabdoid tumor	8 (19.5)
Germ cell tumor	3 (7.3)
Embryonal tumor	2 (4.9)
Disease type, *n* (%)	
New onset	20 (48.8)
Relapse	21 (51.2)
Prior HSCT transplantations, *n* (%)	
0	35 (85.4)
1	4 (9.8)
≥2	2 (4.9)
Complications^b^, *n* (%)	33 (80.5)
Constipation	14 (34.1)
Dry skin	4 (9.8)
Deafness	3 (7.3)

All patients were Asian.

*BSA* body surface area, *ECOG* Eastern Cooperative Oncology Group, *HSCT* hematopoietic stem cell transplantation, *PS* performance status.

^a^Calculated using the Mosteller formula [47] for patients aged <16 years ([(weight [kg] × height [cm])/3600]^½^) or the DuBois formula [48] for those aged ≥16 years (weight [kg]^0.425^ × height [cm]^0.725^ × 0.007184).

^b^Complications occurring in ≥5% of patients are shown.

### Treatments

Dosing frequency and dose administered for thiotepa and melphalan are shown in Supplementary Table S1. All patients (100%) received at least three doses of thiotepa and 39 (95.1%) received all four scheduled doses. All patients (100%) received at least two doses of melphalan and 32 (78.0%) received all three scheduled doses.

### Safety

TEAEs occurring in ≥10% of patients are shown in Table 2. The most frequently reported TEAEs (in ≥50% of patients) were diarrhea (40/41 [97.6%]), febrile neutropenia (34/41 [82.9%]), vomiting (31/41 [75.6%]), stomatitis (26/41 [63.4%]), and nausea (21/41 [51.2%]). Grade III febrile neutropenia was reported in 34/41 (82.9%) patients. The majority of other TEAEs were grade I or II in intensity.

**Table 2:** Summary of TEAEs (safety analysis set).

MedDRA preferred term	Patients with pediatric solid tumors or brain tumors (*N* = 41)	
	All grades	Grade III or IV
Any TEAE, *n* (%)	41 (100.0)	40 (97.6)
TEAEs occurring in ≥10% of patients, *n* (%)		
Diarrhea	40 (97.6)	8 (19.5)
Febrile neutropenia	34 (82.9)	34 (82.9)
Vomiting	31 (75.6)	3 (7.3)
Stomatitis	26 (63.4)	14 (34.1)
Nausea	21 (51.2)	3 (7.3)
Abdominal pain	14 (34.1)	2 (4.9)
Decreased appetite	13 (31.7)	8 (19.5)
Hepatic function abnormal	13 (31.7)	3 (7.3)
Rash	13 (31.7)	0
Malaise	12 (29.3)	0
Skin hyperpigmentation	10 (24.4)	0
Alanine aminotransferase increased	8 (19.5)	0
Aspartate aminotransferase increased	8 (19.5)	0
Edema	8 (19.5)	0
Epistaxis	8 (19.5)	0
Pruritus	8 (19.5)	0
Hypoalbuminemia	7 (17.1)	3 (7.3)
Dry skin	6 (14.6)	1 (2.4)
Face edema	6 (14.6)	0
Pyrexia	6 (14.6)	0
Antithrombin III decreased	5 (12.2)	0
Device-related infection	5 (12.2)	5 (12.2)
Hematuria	5 (12.2)	1 (2.4)

No grade V events were reported.

*MedDRA* Medical Dictionary for Regulatory Activities, *TEAE* treatment-emergent adverse event.

No TEAE resulting in death or treatment discontinuation/dose reduction was reported during the study. TEAEs requiring treatment interruption were observed in 2/41 (4.9%) patients. One patient had an event of bacteremia (this developed on HSCT day −9 and recovered on day 16 post-HSCT) and the other presented with herpes zoster (this developed on day −6 and recovered on day 14 post-HSCT); neither event was considered to be related to the study treatment. In both patients, the scheduled thiotepa dose on day −5 was missed although the other scheduled doses were administered.

Three patients reported serious TEAEs (sepsis grade IV, *n* = 1; tumor hemorrhage grade III, *n* = 1; bacterial enteritis grade IV, *n* = 1). The sepsis was considered unrelated to the study treatment (it was thought to be associated with neutropenia), while the other two events were considered possibly related to treatment. All events were reported as resolved/recovered at the end of the study.

ECOG PS at day 28 post-HSCT remained at 0 in 27 patients (65.9%), and was 1, 2, and 3 in 11 (26.8%), 2 (4.9%), and 1 (2.4%), respectively. No clinically significant change in left ventricular ejection fraction, vital signs or weight was noted during the study.

### Efficacy

Efficacy outcomes are summarized in Table 3. All 41 patients (100.0%) experienced bone marrow suppression and 39/41 (95.1%) achieved engraftment. The median time to engraftment in those 39 patients was 11.0 days. The remaining two patients did not meet the narrow definition of engraftment used in this study (neutrophil count ≥500/mm^3^ for 3 consecutive days), but both patients did achieve a neutrophil count ≥500/mm^3^ on multiple non-consecutive days after autologous HSCT (Supplementary Table S2). The median (range) follow-up after autologous HSCT was 101.0 (100–129) days. The survival rate in evaluable patients at day 100 post-HSCT was 100% (Supplementary Fig. 1).

**Table 3:** Summary of efficacy outcomes (safety analysis set).

	Patients with pediatric solid tumors or brain tumors (*N* = 41)
Bone marrow suppression, *n* (%) [95% CI]	41 (100.0) [91.4, 100.0]
Neutrophil engraftment^a^, *n* (%) [95% CI]	39 (95.1) [83.5, 99.4]
Time to engraftment (days), median (min, max)	11.0 (9, 23)
Evaluable subjects at day 100 post-HSCT, *n*	41
Survival at day 100, *n* (%) [95% CI]	41 (100.0) [-, -]

*CI* confidence interval, *HSCT* hematopoietic stem cell transplantation.

^a^Defined as the proportion of patients with a neutrophil count ≥500/mm^3^ for 3 consecutive days after bone marrow suppression and HSCT.

## Discussion

In this expanded access study, we evaluated the use of thiotepa HDT, in conjunction with melphalan, in Japanese patients with pediatric solid and brain tumors who underwent autologous HSCT. The treatment regimen was found to be tolerable. No unexpected safety outcomes were reported, and there were no discontinuations or deaths due to TEAEs during the study.

The most frequently reported TEAEs in this study were gastrointestinal toxicity and febrile neutropenia, which are commonly associated with chemotherapeutic treatment of pediatric patients [19–22]. The data were also consistent with those reported in pediatric patients with solid tumors in the phase I study [8], and with the European product label [13] and Japanese package insert [17]. Events of veno-occlusive disease of the liver [23], thrombotic microangiopathy [24], and neurotoxic complications [25, 26], which are commonly reported during pre-HSCT treatment, were not observed in this study population.

In early studies of thiotepa as HDT with autologous HSCT, several problematic and/or dose-limiting toxicities were reported, including mucositis and neurotoxicity [27, 28]. Various regimens have since been evaluated, using combinations of thiotepa plus other alkylating agents, such as melphalan and busulfan [29, 30], in an attempt to increase the dose intensity while minimizing the potential for TEAEs. Nonetheless, toxicities remained problematic with many regimens. In the Head Start study, 37 children with malignant brain tumors received carboplatin, thiotepa, and etoposide as HDT, of whom 3 (8%) died of treatment-related complications [31]; however, this rate decreased in the subsequent Head Start II and III trials, with the reduction attributed by the authors to clinician experience with regimen administration and improved procedural and supportive care [32]. In a French study of 116 children who received a busulfan-thiotepa dual HDT regimen, 31% developed veno-occlusive disease [33]. The treatment regimen used in the current study was slightly different from that used in the most recent prior report [30], and decreased the melphalan dose to three-quarters of the prior dose (no infusion on Day -12). This regimen appears to achieve the aim of high myeloablative exposure with few problematic toxicities. Thus, patients in this study experienced a low frequency of pulmonary toxicity and veno-occlusive disease of the liver, which were a common occurrence in HDT regimens containing busulfan, and few events of nephrotoxicity which were previously reported to be associated with HDT regimens including carboplatin, etoposide, and melphalan [34, 35]. Importantly, no patients died due to treatment-related complications. Thus, while we cannot directly compare different thiotepa-containing HDT regimens, we anticipate that the regimen used herein may be less toxic for patients.

Although no renal dysfunction was noted under clinical trial conditions, patients need to be carefully monitored in routine clinical practice, as renal failure has been reported in association with thiotepa [29, 36]. To date, the effects of thiotepa in patients with renal insufficiency have not been assessed, but caution and careful monitoring should be used in patients with a history of renal disease [13].

Clinical efficacy outcomes were positive. All patients in the study experienced bone marrow suppression and all survived for 100 days post-HSCT. The type of solid tumor (brain or other) and number of prior HSCT procedures did not affect the results, and >95% of patients achieved engraftment.

Although radiotherapy is commonly administered to adult patients, irradiation for pediatric CNS tumors comes with an increased risk of later developmental adverse events [37–39]. A recent focus of research has been the use of HDT in combination with radiotherapy to reduce the irradiation exposure necessary for an adequate antitumor effect [40, 41]; the outcomes observed with such combination regimens have been positive. However, HDT agents which are able to transition into the CNS, such as thiotepa, are likely to provide more clinical benefit in this regard. Furthermore, the observation that children tend to tolerate HDT better than adults [42], and the improved outcomes for pediatric primary malignant CNS tumors with the use of HDT and autologous HSCT reported from the ‘Head Start’ trials [31, 43, 44], has led to the administration of HDT plus autologous HSCT as a potential curative option for patients with high-risk disease. Regimens incorporating two alkylating agents are commonly used as HDT prior to HSCT in other malignancies, and have been reported to provide enhanced clinical benefit compared with a single drug [45, 46]. Our results confirm the safety and efficacy data observed in the prior phase I study [8], and thiotepa in conjunction with melphalan appears to be a useful addition to the treatment armamentarium for HDT prior to autologous HSCT in Japanese patients with pediatric solid CNS/non-CNS tumors. Notably, the penetrative ability of thiotepa into the CNS [8, 10] is a key facet supporting its use for this indication.

There are some study limitations that should be considered when evaluating these data. The open-label design and small size of the study restrict the conclusions that can be drawn, and the enrollment of only Asian patients may preclude the extrapolation of the results to the global patient population. Finally, further studies will be necessary to evaluate outcomes over longer durations, as we did not examine long-term survival beyond the first 100 days post-HSCT.

In conclusion, we have confirmed the safety of IV thiotepa HDT, in conjunction with melphalan, prior to autologous HSCT for patients with pediatric solid or brain tumors, with no new toxicity concerns. We consider that thiotepa is a suitable HDT agent for this patient population.

## Supplementary information

Supplemental

Online supplementary material

## Data Availability

The research data underlying this study are subject to restrictions and cannot be shared.
