# Correction: High-dose thiotepa, in conjunction with melphalan, followed by autologous hematopoietic stem cell transplantation in patients with pediatric solid tumors, including brain tumors

**DOI:** 10.1038/s41409-022-01890-5

**Published:** 2022-12-08

**Authors:** Junichi Hara, Kimikazu Matsumoto, Naoko Maeda, Mariko Takahara-Matsubara, Saori Sugimoto, Hiroaki Goto

**Affiliations:** 1 Department of Pediatric Hematology/Oncology, Children’s Medical Center Osaka City General Hospital, Osaka, Japan; 2 Children’s Cancer Center, National Center for Child Health and Development, Tokyo, Japan; 3 Department of Paediatrics, National Hospital Organization, Nagoya Medical Center, Nagoya, Japan; 4 Sumitomo Pharma Co., Ltd, Osaka, Japan; 5 Division of Hematology/Oncology, Kanagawa Children’s Medical Center, Yokohama, Japan

**Keywords:** Drug development, Chemotherapy

Correction to: *Bone Marrow Transplantation* 10.1038/s41409-022-01820-5, published online 03 November 2022

The information to Supplementary Table 2 was given by mistake as Supplementary Table 1 in the sentence: The remaining two patients did not meet the narrow definition of engraftment used in this study (neutrophil count ≥500/mm^3^ for 3 consecutive days), but both patients did achieve a neutrophil count ≥500/mm^3^ on multiple non-consecutive days after autologous HSCT (Supplementary Table S2).

In addition Supplementary Table 1 was missing from this article and has now been uploaded.

The original article has been corrected.

## Supplementary information

Online supplementary material

