# Supplementary material for: High-dose thiotepa, in conjunction with melphalan, followed by autologous hematopoietic stem cell transplantation in patients with pediatric solid tumors, including brain tumors
Source: Bone Marrow Transplant. 2022 Nov 3;58(2):123–8. doi: 10.1038/s41409-022-01820-5 (PMC9902273; doi:10.1038/s41409-022-01820-5)
Supplement: Supplementary file 1 — Supplemental [file 41409_2022_1820_MOESM1_ESM.pdf]

Online supplementary material

Table S1. Neutrophil count by day post-HSCT in two patients who did not meet the criteria for successful engraftment

Neutrophil count (/mm^3) after HSCT	Day 7	Day 10	Day 14	Day 15	Day 21	Day 28
Patient #1	5	-	2 813	-	748	940
Patient #2	0	1 250	6 050	5 650	1 220	1 160

HSCT hematopoietic stem cell transplantation

Fig. S1 Kaplan-Meier analysis of survival after autologous HSCT

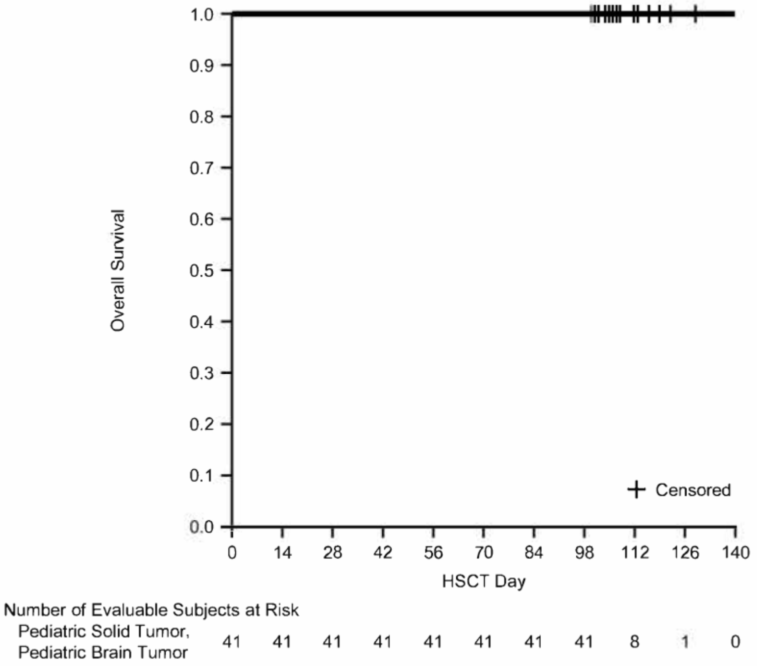

HSCT hematopoietic stem cell transplantation
