# Supplementary material for: High-dose thiotepa, in conjunction with melphalan, followed by autologous hematopoietic stem cell transplantation in patients with pediatric solid tumors, including brain tumors
Source: Bone Marrow Transplant. 2022 Nov 3;58(2):123–8. doi: 10.1038/s41409-022-01820-5 (PMC9902273; doi:10.1038/s41409-022-01820-5)
Supplement: Supplementary file 2 — Online supplementary material [file 41409_2022_1820_MOESM2_ESM.docx]

# Online supplementary material

**Table S1** Treatment details (safety analysis set)

|  | Thiotepa | Melphalan |
| --- | --- | --- |
| Number of exposure days, *n* (%) |  |  |
| 1 | 0 | 0 |
| 2 | 0 | 9 (22.0) |
| 3 | 2 (4.9) | 32 (78.0) |
| 4 | 39 (95.1) | - |
| Daily dose (mg/m^2^/day), median (min, max) | 200 (118, 200) | 70 (30, 70) |
| Cumulative dose (mg/m^2^), median (min, max) | 800 (472, 800) | 210 (90, 210) |
| Dose intensity (%), median (min, max) | 100.0 (59.0, 100.0) | 100.0 (42.9, 100.0) |

**Table S2.** Neutrophil count by day post-HSCT in two patients who did not meet the criteria for successful engraftment

| Neutrophil count (/mm^3^) after HSCT | Day 7 | Day 10 | Day 14 | Day 15 | Day 21 | Day 28 |
| --- | --- | --- | --- | --- | --- | --- |
| Patient #1 | 5 | - | 2 813 | - | 748 | 940 |
| Patient #2 | 0 | 1 250 | 6 050 | 5 650 | 1 220 | 1 160 |

*HSCT* hematopoietic stem cell transplantation

**Fig. S1** Kaplan-Meier analysis of survival after autologous HSCT


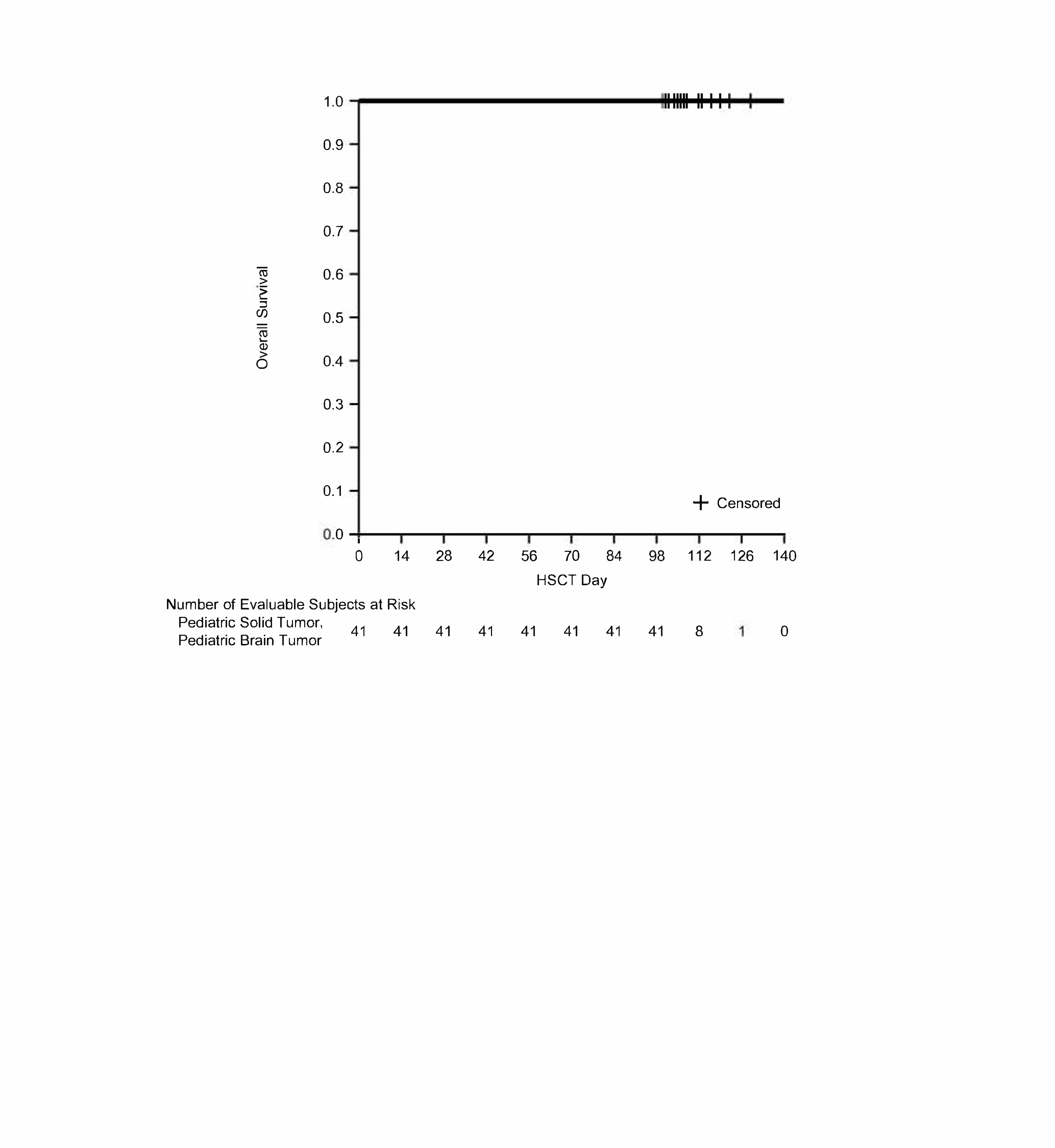


*HSCT* hematopoietic stem cell transplantation
