# Supplementary material for: Correction: High-dose thiotepa, in conjunction with melphalan, followed by autologous hematopoietic stem cell transplantation in patients with pediatric solid tumors, including brain tumors
Source: Bone Marrow Transplant. 2022 Dec 8;58(2):238. doi: 10.1038/s41409-022-01890-5 (PMC9902272; doi:10.1038/s41409-022-01890-5)
Supplement: Supplementary file 1 — Online supplementary material [file 41409_2022_1890_MOESM1_ESM.pdf]

Online supplementary material

Table S1 Treatment details (safety analysis set)

	Thiotepa	Melphalan
Number of exposure days, *n* (%)		
1	0	0
2	0	9 (22.0)
3	2 (4.9)	32 (78.0)
4	39 (95.1)	-
Daily dose (mg/m^2/day), median (min, max)	200 (118, 200)	70 (30, 70)
Cumulative dose (mg/m^2), median (min, max)	800 (472, 800)	210 (90, 210)
Dose intensity (%), median (min, max)	100.0 (59.0, 100.0)	100.0 (42.9, 100.0)

Table S2. Neutrophil count by day post-HSCT in two patients who did not meet the criteria for successful engraftment

Neutrophil count ($/mm^3$) after HSCT	Day 7	Day 10	Day 14	Day 15	Day 21	Day 28
Patient #1	5	-	2 813	-	748	940
Patient #2	0	1 250	6 050	5 650	1 220	1 160

HSCT hematopoietic stem cell transplantation

Fig. S1 Kaplan-Meier analysis of survival after autologous HSCT

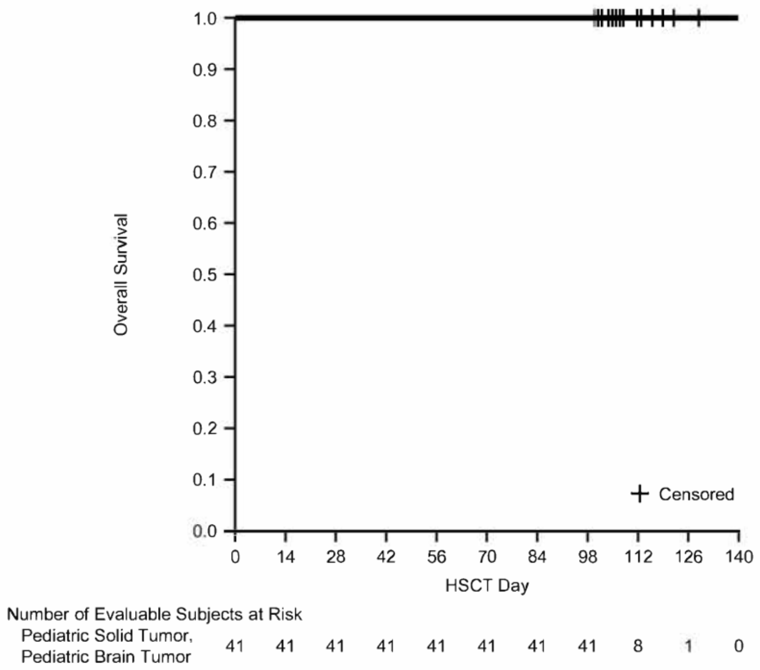

HSCT hematopoietic stem cell transplantation
